# Channel Formation by LktA of *Mannheimia (Pasteurella) haemolytica* in Lipid Bilayer Membranes and Comparison of Channel Properties with Other RTX-Cytolysins

**DOI:** 10.3390/toxins11100604

**Published:** 2019-10-17

**Authors:** Roland Benz, Claudio Piselli, Andrew A. Potter

**Affiliations:** 1 Department of Life Sciences and Chemistry, Jacobs-University Bremen gGmbH Campusring, 1; 28759 Bremen, Germany; c.piselli@jacobs-university.de; 2 Vaccine and Infectious Disease Organization-International Vaccine Centre, University of Saskatchewan, 120 Veterinary Road, Saskatoon, SK S7N5E3, Canada; andrew.potter@usask.ca

**Keywords:** RTX-toxin, cytolysin, channel formation, shipping fever pneumonia, *Mannheimia haemolytica*, lipid bilayer membrane

## Abstract

Cytolysin LktA is one of the major pathogenicity factors of *Mannheimia haemolytica* (formerly *Pasteurella haemolytica*) that is the cause of pasteurellosis, also known as shipping fever pneumonia, causing substantial loss of sheep and cattle during transport. LktA belongs to the family of RTX-toxins (Repeats in ToXins) that are produced as pathogenicity factors by a variety of Gram-negative bacteria. Sublytic concentrations of LktA cause inflammatory responses of ovine leukocytes. Higher concentrations result in formation of transmembrane channels in target cells that may cause cell lysis and apoptosis. In this study we investigated channel formation by LktA in artificial lipid bilayer membranes made of different lipids. LktA purified from culture supernatants by polyethylene glycol 4000 precipitation and lyophilization had to be activated to frequently form channels by solution in 6 M urea. The LktA channels had a single-channel conductance of about 60 pS in 0.1 M KCl, which is about one tenth of the conductance of most RTX-toxins with the exception of adenylate cyclase toxin of *Bordetella pertussis*. The LktA channels are highly cation-selective caused by negative net charges. The theoretical treatment of the conductance of LktA as a function of the bulk aqueous concentration allowed a rough estimate of the channel diameter, which is around 1.5 nm. The size of the LktA channel is discussed with respect to channels formed by other RTX-toxins. We present here the first investigation of LktA in a reconstituted system.

## 1. Introduction

*Mannheimia* (formerly *Pasteurella*) *haemolytica* is a Gram-negative, facultative anaerobic bacterium of the family Pasteurellaceae within the class Gammaproteobacteria [1]. This pathogenic bacterium is responsible for the economically important loss of cattle and sheep during transport that can account for up to 1 million US$ per year [2,3]. The bacterium causes respiratory tract infections lethal for ruminants that are known as pneumonic pasteurellosis or as “shipping fever” [4,5]. So far, it has been demonstrated that the pathogen consists of genetically distinct subpopulations that cause pasteurellosis in cattle or sheep [6,7]. Different strains of *M. haemolytica* have been characterized for the presence of pathogenicity factors [8]. Most of these factors need further evaluation of their role in pasteurellosis, but it is quite clear that lipopolysaccharide (LPS) and leukotoxin (LktA) represent the major pathogenicity factors of *M. haemolytica* [2,5,9,10,11,12,13,14], but also the capsule may play an important role in pathogenesis [15,16].

Considering all the possible pathogenicity factors it seems that leukotoxin LtkA plays a major role in pasteurellosis [8,12,17,18]. This cytolysin belongs to the continuously emerging family of RTX-toxins that comprise more than 1000 species exhibiting various specific biological activities [19]. Typical for the RTX-toxins (Repeats in ToXin) is a long stretch of hydrophilic amino acids in the C-terminal half of the toxin proteins. This domain contains a variable number of glycine-rich and aspartate-containing nonapeptide repeats of the consensus sequence G-G-X-G-(N/D)-D-X-(L/I/F)-X (where X can be any amino acid) [20,21]. The primary sequence of LktA studied here (accession number WP_006248023.1) contains five repeats between amino acid 734 and 787 [10,22,23]. The repeat domain of LktA and the other RTX-toxins binds calcium followed by the formation of β-roll motifs [24]. Calcium binding is essential for biological activity of LktA but probably not for its channel formation in artificial lipid bilayers, similar to the situation for HlyA of *E. coli* because it has been shown for HlyA that it forms also channels when the calcium concentration is extremely low [25]. The gene coding for LktA, *lktA*, is part of an operon that comprises also three additional genes in the series *lktCABD* (in transcriptional order) [10,22,23]. The *lktC* gene codes for a lysine-acyltransferase protein that is required for posttranslational activation of the LktA protoxin prior to secretion. The genes coding for LktB and LktD are required for secretion of the toxin from the organism [26]. They form together with a TolC-homologous protein of the outer membrane of *M. haemolytica* a Type I export system that is driven by ATP-hydrolysis through LktB, which is a member of the ATP-binding cassette superfamily of transport proteins. The membrane-fusion protein LktD provides the contact between the TolC-like channel protein of the outer membrane [27,28,29] and LktB of the inner membrane and is an essential part of the Type I export system [30]. The export signal for the transport of LktA out of the cells is located in its C-terminus similar to other RTX-toxins and comprises about 50 amino acids from the C-terminal end of the RTX-toxins [31].

LktA appears to be specific for ruminant lymphoid cells [32,33]. Important for its interaction with target cells is the presence of a cell surface exposed receptor, which is provided by bovine beta2 integrin LFA-1 in target cells such as bovine alveolar macrophages [34,35]. This has been shown by recombinant expression of ovine LFA-1 on the cell surface of the human erythroleukemic K562 cell line [36]. LFA-1 serves as the functional leukotoxin receptor on ruminant leukocytes. This binding is calcium dependent, which has to do with the formation of the β-roll motifs [24], which are substantial for biological activity of the RTX-toxins. At low, i.e., sublytic, concentrations, LktA induces bovine cells to undergo a respiratory burst and acts as virulence factor in inducing the release of inflammatory mediators and cytokine production. At higher, i.e., lytic, concentrations, LktA induces obviously the formation of transmembrane pores in the cytoplasmic membrane of the macrophages that may lead to apoptosis and/or cytolysis of the cells [37,38,39]. The pores are likely relatively small because sucrose was able to inhibit LktA-induced swelling of BL-3 B lymphocyte cells when they were immersed in a medium made hypertonic by inclusion of 75 mM sucrose. Interestingly, mannose was not able to block swelling. Thus, it was concluded that the diameter of the LktA channels was about 0.9 nm, close to the size of sucrose [37].

In this study, we investigated channel formation by LktA in artificial membranes made of diphytanoyl phosphatidylcholine/n-decane in detail using electrophysiological methods. LktA forms in these membranes and in membranes formed of asolectin/n-decane channels with a relatively low single-channel conductance, much smaller than those measured previously for other RTX-toxins with the exception of CyaA (ACT) of *Bordetella pertussis* [40,41,42]. The LktA channel is highly cation‑selective because of negatively charged groups in or near the channel and responds only moderately to high voltages. The presence of net negative charges allows a rough estimate of the channel diameter, which is about 1.5 nm, somewhat larger than the size of sucrose [37]. This result may indicate that the diameter of the LktA channel may have been underestimated from the swelling of the BL-3 lymphocyte cells treated with LktA because the exclusion limit of the LktA channel may be close to 500 Da, slightly higher than the molecular mass of sucrose (342 Da) [37].

## 2. Results

### 2.1. Purification of LktA

**Figure 1:**
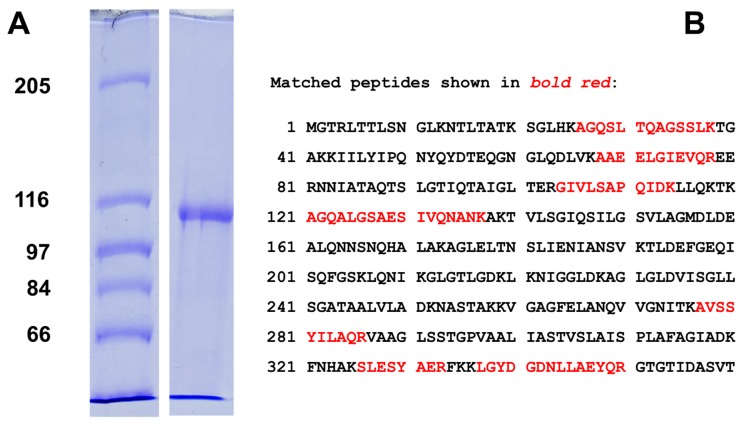
(**A**) 7.5% SDS–PAGE of purified LktA of *M. haemolytica*. The left lane shows molecular mass markers: 205 kDa, 116 kDa, 97 kDa, 84 kDa, and 66 kDa. The right lane shows LktA purified according to the method described in experimental conditions with an apparent molecular mass around 100 kDa. The gel was stained with Coomassie brilliant blue. (**B**) Results of mass spectrometry of a 105-kDa band from SDS–PAGE of LktA. The 105-kDa band of an SDS–PAGE similar to Figure 1A was excised and subjected to trypsin treatment followed by mass spectrometry. The protein was identified as LktA from *M. haemolytica* with the accession number WP_006248023.1. The peptides that matched parts of the first 360 amino acids of LktA are shown in red.

An overnight culture of *E. coli* AA525 was diluted 100‑fold in the growth medium and was cultivated further until an OD_660_ of 1.0 was reached. The supernatant of this culture contained LktA that was precipitated with PEG 4000 followed by centrifugation. The LktA containing pellet was dissolved in Tris-buffered saline (TBS) at pH 7.5, followed by two‑fold dialysis with the same solution. The LktA solution was frozen and lyophilized. Purified LktA was essentially free of contaminant proteins as shown in Figure 1A by a 7.5% SDS-PAGE.

To verify that the 105 kDa band in the SDS–PAGE of Figure 1A is indeed LktA a 105 kDa band from a similar SDS–PAGE was subjected to trypsin treatment followed by mass spectrometry. Nineteen different peptides were identified as partial peptides of LktA of *M. haemolytica* with the accession number WP_006248023.1 as it is shown in Figure 1B for 360 amino acids of LktA. The results of the mass spectrometry verified that we were indeed here working with an LktA variant of *M. haemolytica*.

### 2.2. LktA Obtained by Polyethylene Glycol 4000 Precipitation Had an Extremely Low Membrane Activity

**Figure 2:**
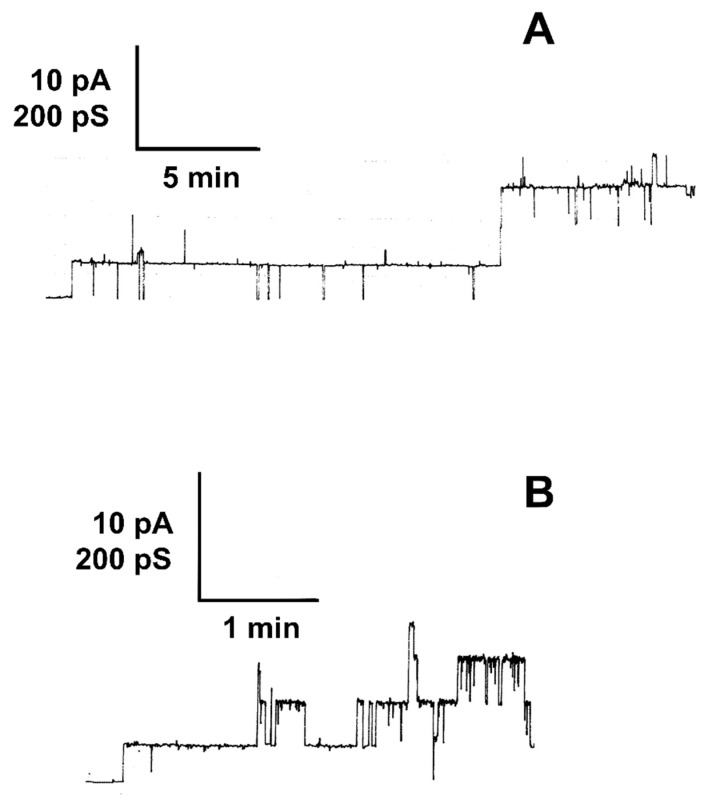
Single-channel recordings of DiPhPC/*n*-decane membranes in presence of pure LktA of *M*. *haemolytica* dissolved in distilled H_2_O (**A**) or treated with 6 M urea (**B**). (**A**) The aqueous phase contained unbuffered 0.1 M KCl, pH 6.0, and about 0.2 µg/mL LktA dissolved in ultrapure H_2_O. The applied membrane potential was 50 mV and the temperature was 20 °C. (**B**) The aqueous phase contained unbuffered 0.1 M KCl, pH 6.0, and about 0.04 ng/mL LktA treated with 6 M urea. The applied membrane potential was 50 mV and the temperature was 20 °C.

In a first set of experimental conditions, we studied the membrane activity of LktA that was obtained by precipitation with polyethylene glycol 4000 (PEG 4000) followed by lyophilization with black DiPhPC/n-decane membranes. This lipid was chosen because of the high stability of lipid bilayers and its zwitterionic nature, which means that it should not interfere with a possible selectivity of LktA. Surprisingly the cytolysin dissolved in H_2_O had no detectable membrane activity and even at very high toxin concentration the membrane conductance did not increase remarkably above that of ground level conductance (10^−7^ S/cm^2^). Similarly, we did not observe increased membrane conductance when membranes made of asolectin (a lipid mixture isolated from soy beans with the neutral phosphatidylcholine and phosphatidylethanolamine as major components) were used, which normally represent an excellent target for channel formation by RTX-toxins [43,44,45]. We checked also the possibility that very high concentration of LktA dissolved in water resulted in observation of current fluctuations in at very high current resolution. Figure 2A shows an experiment of this type. 1 µg of LtkA dissolved in 20 µl H_2_O was added to the 5 mL 0.1 M KCl solution on one side of a black lipid bilayer membrane made of DiPhPC/n-decane. After waiting for 20 min, we observed some current fluctuations of about 60 pS conductance as shown in Figure 2A. These fluctuations were very rare, which means that it was impossible to perform any statistics with the few channels observed under these conditions.

The lack of activity could be caused by misfolding and/or aggregation of LktA. Therefore, we used a protocol that allowed the dissociation of putative aggregates or could promote the water-soluble structure of LktA, similar as in the case of ACT of *Bordetella pertussis* [46]. The membrane activity of this RTX-toxin was preserved when it was kept in 8 M urea and highest membrane activity was observed when it was added in 8 M urea directly to the aqueous phase in membrane experiments [40,47]. A similar observation was made for HlyA of *E. coli* where solution of the hemolysin in 6 M urea increased its specific activity by more than 100‑fold [48]. Five µg of the lyophilized toxin were dissolved in 6 M urea to see if a similar effect was observed with LktA, before it was added to the aqueous phase bathing a lipid bilayer membrane. Interestingly, this treatment had a strong influence on membrane activity of LktA and the membrane conductance increased many orders of magnitude above that of the ground level conductance of lipid bilayer membranes. Control experiments revealed that this effect was caused by the treatment of the lyophilized LktA with urea and had nothing to do with a direct effect of urea on the lipid bilayers because the concentration of urea was at maximum around 20 mM in the experiments, which had no influence on the membranes. In previous studies, we could demonstrate that the macroscopic conductance in the presence of different RTX-toxins showed an exponential dependence on toxin concentration, indicating the contribution of several toxin monomers to a putative conductive oligomer [43,44]. It has to be noted that a similar effect was not observed for LktA, which means that the macroscopic conductance did not provide evidence for the formation of LktA oligomers.

### 2.3. LktA Dissolved in 6 M Urea Formed Small Ion-Permeable Channels

**Figure 3:**
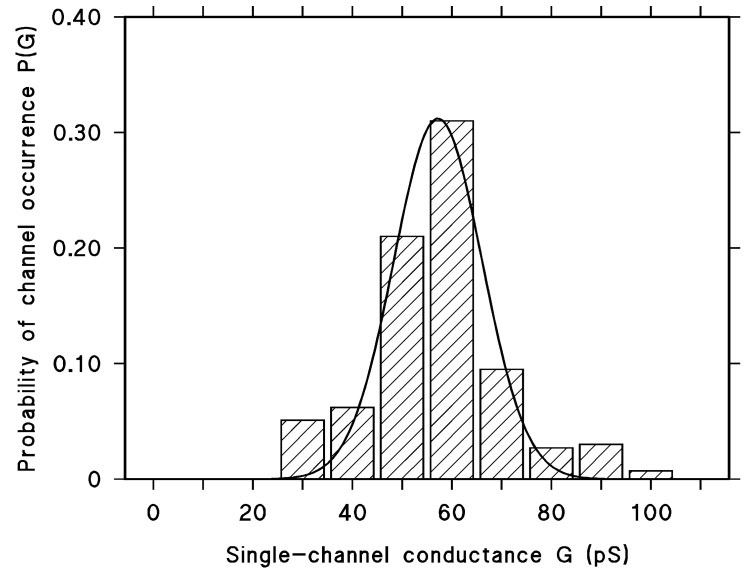
Histogram of the probability P(G) for the occurrence of a given conductivity unit in DiPhPC/*n*-decane membranes in the presence of LktA of *M. haemolytica* treated with 6 M urea. The most frequent single-channel conductance (G in pS) was 60 pS for 78 single-channel events collected from different individual membranes. Aqueous phase contained 0.1 M KCl, pH 6, and about 0.04 ng/mL LktA. The applied voltage was 50 mV and the temperature was 20 °C. The solid line shows a fit of the histogram with a Gaussian distribution. The maximum of the distribution is at a probability of 0.31 ± 0.02 and the conductance is 57 ± 9 pS for 243 single events in total taken from 12 individual membranes, V_m_ = 50 mV, T = 20 °C.

Many cytolysins of the RTX-family form ion-permeable channels [42]. To investigate whether this was also the case for LktA treated with urea we studied its membrane activity at high current resolution. Figure 2B shows an experiment of this type at a salt concentration of 0.1 M KCl, pH6. LktA was added in a final concentration of about 0.04 ng/mL to one side of a black lipid bilayer membrane. Shortly after addition, the membrane current increased in a step-like fashion indicating the formation of small ion-permeable channels. Figure 2A demonstrates that the channels had a limited lifetime of about 10 s but it was longer than that observed previously for HlyA of uropathogenic *E. coli* [43] or the different Apx-toxins of *Actinobacillus pleuropneumoniae* [45]. The single-channel conductance was, on the other hand, with about 60 pS in 0.1 M KCl much smaller than that of most channel-forming RTX-toxins studied to date [41,42,43,44,45]. Only ACT of *B. pertussis* forms even lower conductive channels (10 pS at 0.1 M KCl, pH 7 [47]). The channels formed by LktA in DiPhPC/n-decane membranes were fairly homogeneous as the histogram of 243 channels shown in Figure 3 indicates. A Gaussian fit of all conductance fluctuations yielded an average single-channel conductance of 57 ± 9 pS.

### 2.4. Results of Single-Channel Analysis

**Figure 4:**
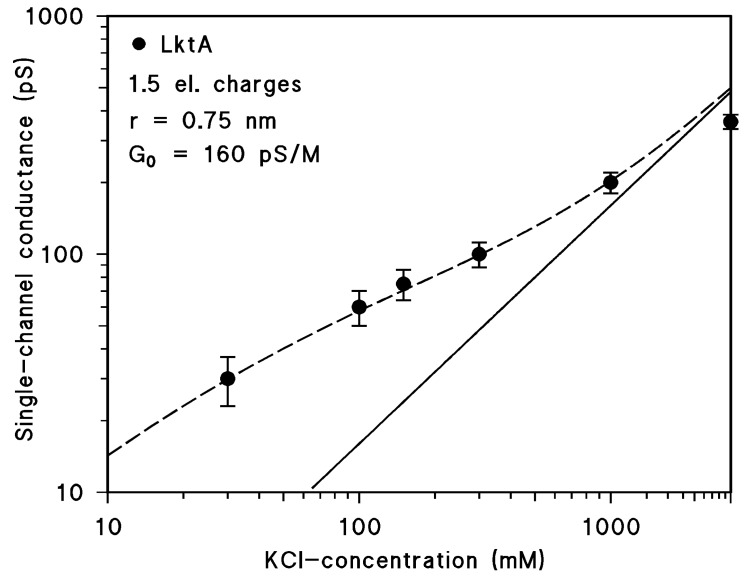
Single-channel conductance of LktA of *M. haemolytica* as a function the KCl-concentration in the aqueous phase (filled circles). The broken line represents the fit of the single-channel conductance data with Equations (2) to (4) assuming the presence of negative point charges (1.5 negative charges; q = –2.4 × 10^–19^ As) at the channel mouth and assuming a channel diameter of 1.5 nm of the LktA channel. c, concentration of the KCl solution in M (molar); G, average single-channel conductance in pS (pico Siemens, 10^−12^ S). The solid (straight) line shows the single-channel conductance of LktA that would be expected without point charges. It corresponds to a linear graph between channel conductance and bulk aqueous concentration at a double logarithmic scale.

We extended the single-channel analysis of LktA to other conditions and changed the KCl-concentration and the composition of the aqueous salts. In addition, we checked also channel-formation in asolectin membranes. The LktA channels had in asolectin membranes the same conductance as in DiPhPC/n-decane membranes. The cytolysin formed also channels in 1 M LiCl (pH 6) and 1 M KCH_3_COO (pH 7) solutions with about the same frequency as in 1 M KCl. The average conductance (summarized in Table 1) was in 1 M LiCl less than one half of that in 1 M KCl, whereas the conductance was approximately the same in 1 M KCH_3_COO. When these results were compared with the limiting conductivity of the different anions and cations in the aqueous phase, which is a measure of their aqueous mobilities (also given in Table 1), then it is clear that the LktA channel preferred cationic solutes because its conductance followed the mobility of the cations. We performed also single-channel conductance measurements in different KCl-concentrations. The channel-forming activity was about constant for concentrations down to 100 mM KCl, but for smaller concentrations, it dropped down considerably indicating a certain influence of the ionic strength on the reconstitution rate of LktA. The data of Table 1 demonstrate that the single-channel conductance was not a linear function of the bulk aqueous concentration. Instead, it looked like if the single-channel conductance is a function of the square root of the concentration because an increase of the concentration by a factor of 100 resulted in an only 10‑fold increase of conductance (see Table 1 and Figure 4).

### 2.5. Selectivity of the LktA Channel

The data of the single-channel conductance measurements indicated already that LktA could form cation‑selective channels because of its larger conductance in KCH_3_COO as compared to LiCl. To verify this observation in detail we performed zero-current-membrane potential measurements with all three salts and 5‑fold gradients (0.1 versus 0.5 M). For all of them the more dilute side (0.1 M) turned positive because of preferential movement of cations through the LktA channel. The zero-current potentials were used to calculate the permeability ratios for the three different salts using the Goldman–Hodgkin–Katz equation given by Equation (1) [50]. Table 2 summarizes the results of the measurements. It demonstrates that the LktA channel was indeed cation‑selective for all three salts. The permeability ratio P_cation_/P_anion_ varied somewhat for them, which may indicate that the anions had also a small permeability through the LktA channel. However, it is also clear from the data of Table 1 and Table 2 that the contributions of the anions to channel conductance was extremely small.

## 3. Discussion

### 3.1. Purification of LktA from E. coli AA525

The *E. coli* strain AA525 contains two plasmids. One codes for the *lkt* gene cluster and the other one is coding for the genes *hlyB* and *hlyD* of the transport function of HlyA of *E. coli*. Involved in export of LktA is also the outer membrane protein TolC that forms together with the ATP-binding cassette protein HlyB/LktB and the membrane fusion protein HlyD/LktD the export apparatus that secretes LktA from the cytosol over two membranes to the supernatant of the *E. coli* cells [28,29]. Prior to export, LktA has to be activated posttranslationally by the lysine-acyltransferase protein LktC. The activation of LktA and other RTX-toxins (i.e., the fatty acid acylation of the two lysines) is not directly involved in channel formation but it is essential for biological activity and enhances also greatly channel formation in lipid bilayer membranes [43,51]. Pure LktA was obtained by its precipitation from the culture supernatant by addition of PEG 4000. The protein was obtained by centrifugation followed by solution of the pellet in Tris-buffered saline and lyophilization. LktA obtained by this procedure was essentially pure as shown by SDS–PAGE (Figure 1A). It exhibited biological activity after prolonged incubation times, but it was essentially inactive in the lipid bilayer assay. Mass spectrometry (see Figure 1B) identified the version of LktA used in our study as NCBI Reference Sequence WP_006248023.1, submitted March 22, 2015 to the gene bank.

### 3.2. Single-Channel Analysis

Channel-forming activity increased strongly when the lyophilized material was dissolved in 6 M urea instead of ultrapure water. Similar observations were made previously for ACT (CyaA) of *B. pertussis* [46] and RtxA of *K. kingae* [41]. Similarly, HlyA of *E. coli* showed an increased specific activity when it was dissolved in 6 M urea [48]. In all these cases the single-channel properties were not influenced by the treatment of the RTX-toxins by urea and this was also the case here because the single-channel conductance of LktA in 0.1 M KCl was not changed by the treatment with urea (see Figure 2) [41,46,48]. Urea and ions of the Hofmeister series destroy the secondary and tertiary structure of proteins but do not interfere with their primary structures. It seems here that LktA became aggregated during the isolation and purification process, i.e., by precipitation and lyophilization. Urea treatment dissolved presumably the aggregates and channels could then be formed from the water-soluble monomers.

The pore-forming activity of LktA treated with 6 M urea was sufficiently high enough to allow good statistical analysis of all single-channel measurements similar as shown in Figure 3 for 0.1 M KCl. Interestingly, the single-channel conductance of the LktA channel was much smaller than those of the hemolytically active RTX-toxins of *E. coli* [43,52], the *Morganella* group [44], and ApxI of *Actinobacillus pleuropneumoniae* [45]. These hemolysins had all a single-channel conductance around 500 pS in 150 mM KCl, whereas LktA formed channels under the same conditions with a single-channel conductance of about 75 pS, about 7‑times smaller. The reason for this difference is not quite clear because the secondary, tertiary, and quaternary structures of the RTX-channels are not known. Mutations of the RTX-toxins suggested that they contain both α-helical and β-sheet structures [53,54,55]. However, which one of both structures is predominant in pore formation remains open. Important for the formation of the pores is definitely the formation of oligomers that has been shown for RTX-toxins of different Gram-negative pathogens [40,43,56,57]. Oligomerization for LktA could not be demonstrated here, which means presumably that the kinetics of formation of putative conductive LktA-oligomers from monomers was quite slow, such that it was impossible to follow oligomerization on the time scale of electrophysiological experiments.

### 3.3. Negative Point Charges Control Ion Transport through LktA Channels

The conductance of the LktA channels was approximately a function of the square root of the bulk aqueous KCl-concentration (see Table 1). We could show in previous studies that this is the result of net negative charges in or near the channel [43,58]. The same formalism could be used to fit the conductance of the LktA channels as a function of the bulk aqueous KCl-concentration (see Figure 4). The fit yielded two parameters, the number of (excess) negative charges in or near the channel and the diameter of the channel. The number of net charges has to be considered as tentative because the dielectric constant of their environment in or near the channel may vary considerably from ideally being two (i.e., a pure lipid bilayer). This has a big impact on the number of charges [58]. The size of the channel with 1.5 nm appears to be more precise because it has to do with the decay of the potential created by the net charges in the aqueous phase, which is controlled by the Debeye length.

### 3.4. Comparison with Channel Properties of Other RTX-Toxins

We could clearly demonstrate here that LktA of *M. haemolytica* is a channel-forming component. Channel-formation of RTX-toxins is definitely the basis of their biological activity [42]. All the other processes involved in intoxication of target cells, i.e., binding to a surface receptor, calcium-dependent formation of β-roll motives from the repeats and activation of the RTX-toxins by acylation of the two lysines in their N-terminal parts are prerequisites for channel formation as the key process. If one of these processes is disturbed, intoxication of target cells is impaired. Channel formation leads to dissipation of the ionic gradients across the cytoplasmic membranes, which triggers lysis of red blood cells as well as apoptotic processes in immune cells. Even the formation of a small number of pores may be sufficient to start inflammatory disease processes such as respiratory bursts and the release of inflammatory mediators and cytokine production in target cells [32,33,34,35,59].

It has to be noted that channel formation by RTX‑toxins in lipid bilayer membranes is not or only little dependent on the prerequisites listed above. Only the acylation of HlyA of *E. coli* at the lysines Lys-564 and Lys-690 leads to an enhanced interaction between lipid monolayers and bilayers but has definitely no influence on channel properties [60,61]. Similarly, acylation of CyaA (ACT) from *B. pertussis* at Lys-860 and/or Lys-983 had no or an only negligible effect on its single-channel conductance [51]. We can assume that this is also the case for other RTX‑toxins. So what is the difference between reconstitution of RTX‑toxins in lipid bilayers and the intoxication of target cells? Lipid bilayer membranes have a smooth surface that has no surface structure and allows direct access and interaction of the RTX‑toxins. Biological membranes have surface structures that are composed of connective tissue and carbohydrates. Surface receptors and calcium binding to the repeats boost the presence of RTX‑toxin on the surface of the cytoplasmic membranes and promote the formation of ion-permeable channels, which are the primary event leading to cell lysis and other cellular processes including apoptosis.

Table 3 shows a comparison of the pore-forming characteristics of different RTX-toxins in lipid bilayer membranes but also in biological membranes. The LtkA channel shares with most of them the presence of net negative charges in or near the channel that results in high cation selectivity. The most remarkable difference to the pores formed by many of the other toxins is the size of the LktA channel. Its diameter is together with that of ACT (CyaA) of *B. pertussis* the smallest size of pores/channels formed by RTX-toxins as derived from the fit of the single-channel conductance. It is noteworthy that the diameter of these channels was also derived using a cell-based assay [37]. LktA-induced swelling of BL-3 B lymphocyte cells was inhibited when they were immersed in a medium made hypertonic by inclusion of 75 mM sucrose but not of mannose. This suggested that the diameter of the LktA channels was about 0.9 nm, because the radius of sucrose is close to 0.5 nm [37]. When such a diameter is compared with that derived from the fit of the single-channel data with the net negative charge formalism and the diameters of the pores formed by the other RTX-toxins of Table 3 then the size of the LktA channels formed in the BL-3 B lymphocyte cells appears to be underestimated. This has presumably to do with time effects, as we could demonstrate in a study with deletion mutants of HlyA, which have a smaller single-channel conductance than wt. HlyA [55]. Nevertheless, it appears to be clear from the pore formation in vitro studied here and the result of the cell-based assay that the size of the LktA channel is smaller than that of the pores formed by the RTX-hemolysins [37].

## 4. Materials and Methods

### 4.1. Bacterial Strains, Plasmids, and Culture Conditions

The *Escherichia coli* strain used for LktA production was AA525 that corresponds to *E. coli* LE392 (Promega, Germany), which is an *E. coli* K-12 strain. *E. coli* K-12 5K have frequently been used for the heterologous expression of different RTX-toxins [25,40,43,45,47,48,55,56]. *E. coli* AA525 contains the plasmids pAA210 (a construct based on pHC79 containing the *M. haemolytica* gene cluster, *lktC*, *lktA*, *lktB*, and *lktD*) and pWAM716 (based on pACYC184 coding for the HlyB and HlyD transport function of HlyA of *E.coli*) to ensure LktA export [65]. *E. coli* AA525 was grown in M9 minimal medium supplemented with 0.002% casamino acid, 0.5 µg/mL tryptophan, 50 µg/mL ampicillin, and 25 µg/mL chloramphenicol.

### 4.2. Isolation and Purification of LktA

An overnight culture of *E. coli* AA525 was diluted 100‑fold in 500 mL of the same medium and was grown during vigorous shaking at 37 °C until an OD_660_ of 1.0 was reached. Cells were harvested by centrifugation at 6000 rpm (GSA rotor; Sorvall, Thermo Fisher Scientific, 168 Third Avenue Waltham, MA USA 02451). The supernatant was sterile filtered by passing through 0.2-micron membrane and was chilled on ice for 15 min. Solid polyethylene glycol with a molecular mass of 4000 (PEG 4000; Sigma‑Aldrich; Merck, Darmstadt, Germany) was added to the supernatant in a final concentration of 20% while stirring for 30 min at 4 °C. The solution was then centrifuged at 12,000 rpm (Sorval GSA rotor) and the pellet was dissolved in 5 mL of Tris-buffered saline (TBS) at pH 7.5 followed by dialysis against 2 l of TBS at 4 °C with two changes of TBS. The purified protein was split in five aliquots of one ml that contained about 5 µg LktA each. The aliquots were frozen at −70 °C followed by lyophilization in a Savant SpeedVac (Thermo Fisher Scientific, 168 Third Avenue Waltham, MA USA 02451). Biological activity of LktA was tested using bovine erythrocytes as described elsewhere [66] with a prolonged incubation time of four hours.

### 4.3. SDS‑PAGE

Analytical SDS‑PAGE was performed according to [67]. The gels were stained with Coomassie brilliant blue (Sigma-Aldrich; Merck, Darmstadt, Germany).

### 4.4. Lipid Bilayer Experiments

The methods used for the lipid bilayer measurements have been described previously in detail [68]. The basic part consisted of a Teflon chamber divided into two compartments with volumes of 5 mL each. A small circular hole with a surface area of about 0.4 mm^2^ connected the two compartments. Black lipid bilayer membranes were obtained by painting a solution of 1% (*w*/*v*) diphytanoyl phosphatidylcholine (DiPhPC; Avanti Polar Lipids, Alabaster, AL) [69] or asolectin (phospholipids from soy bean, Sigma‑Aldrich; Merck, Darmstadt, Germany) [70] in n‑decane onto the hole. Asolectin is a lipid mixture from soy beans that contains about 40% neutral phospholipids [70]. LktA was added from a concentrated stock solution (5 µg/mL) to the aqueous phase bathing black lipid bilayer membranes. Due to the extremely low membrane activity of the rehydrated LktA, 5 µg of the lyophilized protein was dissolved in 300 µl 6 M urea. The addition of small amounts of this mixture (5 µl or 10 µl) to black membranes resulted in a rapid increase of conductance. All salts were analytical grade and the temperature was maintained at 20 °C during all experiments. The membrane current was measured with Ag/AgCl electrodes (with 3 M salt bridges) switched in series with a homemade voltage source and a Keithley 427 (Keithley; Cleveland, OH) current amplifier. Zero current membrane potential measurements were obtained by establishing salt gradients across membranes containing about 100 LktA channels using a Keithley 417 electrometer (Keithley; Cleveland, OH) as described elsewhere [48]. The asymmetry potentials, *V_m_*, caused by the salt gradient *c’/c’’* across the membranes were analyzed using the Goldman-Hodgkin-Katz equation [48]:

(1) $$V_{m}=\frac{R\cdot T}{F}\cdot ln\frac{P_{cation}\cdot c^{\prime }+P_{anion}\cdot c^{\prime \prime }}{P_{cation}\cdot c^{\prime \prime }+P_{anion}\cdot c^{\prime }}$$

$P_{cation}\;\mathrm{and}\;P_{anion\;}$ are the permeabilities of the cation and the anion, respectively, R is the ideal gas constant (*R* = 8.31 J⋅K^−1^⋅mol^−1^), *T* the absolute temperature in Kelvins (K), and *F* is the Faraday constant (F = 96,500 A·s/mol). *R·T/F* are about 25.2 mV at 20 °C.

### 4.5. Effects of Point Charges on LktA Conductance

The single-channel conductance of LktA measured for different KCl-concentrations did not follow a linear function between concentration and conductance (see above). There exist two possibilities to explain such a behavior. One is the presence of a binding site for ions, which results in a saturation of conductance for increasing ion concentration. The other possibility is the presence of point charges. Both effects on ion conductance have been discussed in full detail in previous studies [58,71]. Our result suggested that the channel does not contain a binding site for cations and that point negative charges located at the pore mouth cause the cation selectivity. These charges modulate the single-channel conductance by creating a surface potential *Φ*, which is given for a channel with a radius, *r*, and a total net charge, *q* (in A·s) by [58]:

(2) $$\Phi =\frac{2q\cdot e^{-\frac{r}{l_{D}}}}{4\pi \cdot \varepsilon_{0}\cdot \varepsilon \cdot r}$$

where *ε*_0_ (= 8.85 × 10^−12^ F/m) and *ε* (= 80) are the absolute dielectric constant of vacuum and the relative dielectric constant of water, respectively, and *l_D_* is the so called Debeye length, which controls the decay of the potential (and of the accumulated positively charged ions) in the aqueous phase:

(3) $$l_{D}^{2}=\frac{\varepsilon \cdot \varepsilon_{0}\cdot R\cdot T}{2\cdot F^{2}\cdot c}$$

where c is the bulk aqueous salt concentration, *R* is the gas constant, *T* the absolute temperature in Kelvins, and *F* is the Faraday constant (*R·T/F* = 25.2 mV at 20 °C). The concentration of the monovalent cations, $c_{0}^{+}$, at the channel mouth is given by:

(4) $$c_{0}^{+}=c_{0}\cdot e^{-\frac{\Phi \cdot F}{R\cdot T}}$$

The cation concentration $c_{0}^{+}$ at the mouth of the pore can now be used for the calculation of the effective conductance concentration curve. The theoretical treatment provides also some information on the size of the channel and the number of charges involved in ion conductance through the channel.

## Figures and Tables

**Table 1:** Average single-channel conductance of LktA from *M. haemolytica* in different electrolyte solutions and limiting molar conductivities of the cations and anions.

Electrolyte/Ion	Concentration (M)	G (pS)	Limiting Molar Conductivity (S·cm^2^·mol^−1^)
KCl	0.03	30 ± 6	
	0.10	57 ± 9	
	0.15	75 ± 11	
	0.30	100 ± 12	
	1.00	200 ± 20	
	3.00	360 ± 25	
LiCl	1.00	85 ± 11	
KCH_3_COO (pH 7)	1.00	180 ± 18	
K^+^			73.5
Li^+^			38.7
Cl^−^			76.4
CH_3_COO^−^			40.9

The membranes were formed from diphytanoyl phosphatidylcholine dissolved in *n*‑decane. The single‑channel conductance was measured at 50 mV applied voltage and T = 20 °C. The average single‑channel conductance, G (± SD), was calculated from at least 100 single events, similarly to the procedure shown in Figure 3 for 0.1 M KCl. The aqueous mobilities of cations and anions used in the single-channel experiments are given as their limiting molar conductivities in the aqueous phase at 25 °C taken from [49].

**Table 2:** Zero‑current membrane potentials, V_m_, of DiPhPC/*n*‑decane membranes in the presence of LktA of *M. haemolytica* measured for a 10‑fold gradient of different salts ^a^.

Electrolyte	Permeability Ratios P_cation_/P_anion_	V_m_ (mV)
KCl	5.0	31.0 ± 2.4
LiCl	4.6	29.8 ± 1.8
KCH_3_COO (pH 7)	7.3	38.1 ± 2.3

^a^ V_m_ (± SD) was derived from three individual measurements for a given salt. It is defined as the difference between the potential at the dilute side (0.1 M) minus the potential at the concentrated side (0.5 M) of the membranes. The aqueous salt solutions were used unbuffered and had a pH of 6, if not indicated otherwise; T = 20 °C. The permeability ratios P_cation_/P_anion_ were calculated using the Goldman–Hodgkin–Katz equation (Equation (1) [50]) as the mean of at least 3 individual experiments.

**Table 3:** Comparison of pore forming properties of different RTX‑toxins in lipid bilayer membranes.

Toxin	SCC	IS	VG	PD
**LktA**	75 pS	Cation‑select.	No voltage dependence (this study)	1.5 nm (d) (this study)
***M. haemolytica***	(0.15 M KCl) (this study)	P_K_/P_Cl_ = 5.0 (this study)		
**HlyA**	500 pS	Cation‑select. P_K_/P_Cl_ = 9 [43]	<100 mV [43]	1.4–3 nm(b) [62]
***E. coli***	(0.15 M KCl) [43]			
**EHEC-Hly**	500 pS	Cation‑select. P_K_/P_Cl_ = 13 [52]	No data	2.6 nm (b,c) [52]
***E. coli* EHEC**	(0.15 M KCl) [52]			
**RtxA**	400 pS	Cation‑select.	30–40 mV [41]	1.9 nm (a) [41]
***K. kingae***	(0.1 M KCl) [41]	P_K_/P_Cl_ = 3.5 [41]		
**ApxI**	540 pS	Cation‑select.	No data	2.4 nm (c) [45]
***A. pleuropneumoniae***	(0.15 M KCl) [45]	P_K_/P_Cl_ = 5.7 [45]		
**ApxII**	620 pS	Cation‑select.	No data	2.5 nm (c) [45]
***A. pleuropneumoniae***	(0.15 M KCl) [45]	P_K_/P_Cl_ = No data [45]		
**ApxIII**	95 pS	Cation‑select. P_K_/P_Cl_ = 9.6 [45]	No data	1.8 nm (c) [45]
***A. pleuropneumoniae***	(0.15 M KCl) [45]			
**CyaA**	10 pS	Cation‑select. P_K_/P_Cl_ = 9–11 [40]	No data	0.6–0.8 nm (b,c) [40,63]
***B. pertussis***	(0.1 M KCl) [40]			
**Rtx**	500 pS	Cation‑select. (P_K_/P_Cl_ = 9.5) [44]	≥40 mV [44]	2 nm (d) [44]
***P. vulgaris***	(0.15 M KCl) [44]			
**Rtx**	520 pS	Cation‑select. (P_K_/P_Cl_ = 10) [44]	≥40 mV [44]	2 nm (d) [44]
***M. morganii***	(0.15 M KCl) [44]			
**Leukotoxin**	406 pS	No data	No data	No data
***Actinobacillus actinomycetemcomitans***	(0.14 M NaCl, 0.01 M CaCl_2_) [64]			

The biophysical properties such as single-channel conductance (SCC), ion selectivity (IS), voltage-dependent gating (VG), and the pore diameter (PD) are shown for several RTX-toxins. The references to the original studies are included, while the results from this study are indicated by “(this study)”. The SCC showed in the Table refers to the open state of the pore with higher conductance. The P_K_/P_Cl_ values shown in the Table were derived from experiments performed in KCl solutions. The values for the pore diameter were obtained using the following methods; (a) partition experiments with non-electrolytes, (b) osmotic protection experiments, (c) mobility sequence of different cations and, (d) effects of point charges.
